# Trauma patients with metastatic cancer undergoing emergent surgery: A matched cohort analysis

**DOI:** 10.1016/j.sopen.2024.07.005

**Published:** 2024-07-17

**Authors:** Matthew Nguyen, Jeffry Nahmias, Oliver S. Eng, Maheswari Senthil, Cristobal Barrios, Matthew Dolich, Michael Lekawa, Areg Grigorian

**Affiliations:** a University of California, Irvine, Department of Surgery, Division of Trauma, Burns and Surgical Critical Care, Orange, CA, USA; b University of California, Irvine, Department of Surgery, Division of Surgical Oncology, Orange, CA, USA

**Keywords:** Metastatic cancer, Emergent surgery, Palliative care

## Abstract

**Background:**

There is a paucity of literature guiding trauma surgeons in the care of patients with active metastatic cancer (MC). Even less is known regarding outcomes for MC patients requiring emergent surgery after trauma. We hypothesized that trauma patients with active Metastatic Cancer (MC) have an increased mortality rate and undergo increased rates of withdrawal of care (WoC) within 72-hours following emergent operations, compared to similarly matched patients without MC.

**Methods:**

Patients with active MC at the time of traumatic injury were matched 1:2 against patients without active MC based on demographics, comorbidities, vital signs on admission, and injury profile.

**Results:**

From 43,826 patients, 0.2 % had MC. After matching 39 MC patients to 78 without MC, there was no difference in demographics, comorbidities, injury severity score, mechanism of injury, vitals on admission (blood pressure, heart rate, respiration rate) and need for blood transfusion (all *p* > 0.05). Compared to patients without MC, patients with MC had higher rates and associated risk of death during index hospitalization (38.5 % vs. 15.2 %, *p* = 0.005; OR 3.49, CI 1.43–8.51, *p* = 0.006), as well as a higher rate and associated risk of WoC within 72-hours (12.8 % vs. 1.3 %, *p* = 0.007; OR 11.47, CI 1.29–101.93, *p* = 0.029).

**Conclusion:**

Trauma patients with MC requiring emergent thoracic or abdominal surgery have a high risk of death and an over ten-fold higher associated risk for WoC within the first three days. In some cases, palliative care consultation should be considered, and counseling should be offered to this high-risk trauma population to enable individualized and patient-centric decisions.

**Key message:**

This research highlights the importance of a multidisciplinary team consisting of trauma surgeons, oncologist, and palliative care physicians in caring for the high-risk trauma patients with disseminated cancer requiring urgent surgery.

## Highlights

• Patients with metastatic cancer have a higher rate and associated risk of death following emergent operations
• Patients with metastatic cancer have higher rate and risk of withdrawing from care within 72 hours after emergent operations
• Palliative care consultations should be considered for trauma patients with metastatic cancer

## Introduction

There are nearly 2.6 million trauma admissions each year with 11 % requiring operative intervention [1,2]. While most trauma patients are young with minimal comorbidities, nearly 10 % have one or more comorbidities and the severity of comorbidities may be associated with worse outcomes [3]. Wang et al. demonstrated increased in-hospital mortality for trauma patients with a higher index of coexistent comorbidity disease score [4]. The most common comorbidities in trauma patients include diabetes mellitus and hypertension [5,6]. In most patients, particularly with minimal traumatic injuries, these are unlikely to impact clinical outcomes in a significant way [7]. However, certain comorbidities such as chronic obstructive pulmonary disease (COPD) and cirrhosis are associated with substantial increased risk of complications and death in trauma patients [8,9]. In addition, the overall trauma population has mirrored the aging U.S. population, with an estimated 73 million older adults (65 years or older) by 2030 [10]. Although the relationship between most comorbidities and clinical outcomes in trauma patients has been well studied, the impact of concurrent disseminated cancer on trauma patients has not been previously evaluated.

Each year, nearly two million new cancer diagnoses are rendered across the United States and nearly 40 % of these patients will have disseminated disease at the time of presentation [11]. Cancer patients with metastatic disease are particularly susceptible to complications and/or death following trauma given their relatively increased immunocompromised state, deconditioned functional status and hypercoagulable state [[12], [13], [14]]. Cancer patients with metastatic cancer (MC) undergoing emergent surgery may have a higher rate of morbidity and mortality [15,16]. This may also be true for trauma patients. While some of these patients may not be lucid enough following trauma to participate in a detailed discussion regarding the risks and expected outcomes following emergency surgery, this is not the case for all patients [17]. Thus, there may be a role for targeted interventions to reduce complications and suffering by integrating immediate palliative care for these patients which may also help define goals of care [18]. As such, we sought to evaluate clinical outcomes for trauma patients with MC undergoing emergency surgery. We hypothesized that trauma patients with active MC have an increased risk of mortality following emergent thoracic or abdominal operations, compared to similarly matched patients without MC. We additionally hypothesized a higher rate of MC patients undergo withdrawal of care (WoC) within 72-hours, compared to those without MC.

## Methods

This study was deemed exempt by our Institutional Review Board and a waiver of informed consent granted as it utilizes a national deidentified database. A retrospective analysis of the Trauma Quality Improvement Program (TQIP) was performed between 2017 and 2019. TQIP includes over 870 trauma centers across the United States with the goal of improving outcomes for trauma patients [19]. All adult patients (≥18 years) with MC and isolated torso injury undergoing emergent thoracic or abdominal operations were identified. Isolated torso trauma was defined by an abbreviated injury scale (AIS) grade ≤ 1 for the head, spine, upper extremities, and lower extremities. MC is defined by TQIP as patients who have cancer that has spread to one or more sites in addition to the primary site and in whom the presence of multiple metastases indicates the cancer is widespread, fulminant, or near terminal. The location and type of primary cancer or metastatic sites are not included in TQIP. Emergency surgery was defined by any operation on the gastrointestinal, hepatobiliary, urinary, respiratory, or cardiac systems within two-hours of arrival. Patients who were transferred from an outside facility were excluded.

To address the observed imbalance between trauma patients with MC compared to trauma patients without MC, a propensity-matched analysis was utilized. This was derived from a logistic regression model in which the dependent variable was the presence of MC. The covariates utilized in the model included age, sex, comorbidities, injury severity score (ISS), vitals on admission (i.e., Glasgow coma scale score, blood pressure, respiration rate and heart rate), need for packed red blood cell (PRBC) transfusion, and mechanism of injury. The specific comorbidities used for propensity matching included congestive heart failure, COPD, diabetes, hypertension, myocardial infarction, peripheral arterial disease, end-stage renal disease, steroid-use, anticoagulation-use, and smoking. We utilized propensity-score matching to create comparable groups of trauma patients with and without disseminated cancer. Propensity-score matching was performed using a 1:2 matching ratio, where each trauma patient with MC was matched with two trauma patients without MC based on key demographic and clinical variables that were 1) available to us given the limitations of a retrospective database, and 2) were deemed relevant after a discussion amongst coauthors. We employed caliper matching with a caliper width of 0.001 of the estimated logit to ensure precise matching and minimize differences between the groups. This method aims to emulate randomization in observational studies by including only cases within the specified caliper range, thus enhancing the comparability of the matched cohorts.

The primary outcome was mortality, and the secondary outcome was WoC within 72-hours. Other measured outcomes included total hospital length of stay (LOS), ventilator days, and in-hospital complications (acute kidney injury, acute respiratory distress syndrome, cardiac arrest, myocardial infarction, central line associated bloodstream infection (CLABSI), catheter associated urinary tract infection (CAUTI), pneumonia, sepsis, superficial surgical site infection (SSI), deep SSI, deep vein thrombosis, pulmonary embolism, cerebrovascular accident, unplanned intensive care unit (ICU) admission, unplanned intubation, and unplanned return to operating room (OR)).

A bivariate analysis was performed for all variables to confirm a successfully matched cohort. A Chi-square test or a Mann-Whitney *U* test were used to compare categorical variables and continuous variables, respectively. Categorical data were presented as percentages, while continuous data were presented as medians with interquartile range. All *p-*values were two-sided, with a statistical significance level of <0.05. In addition, a logistic regression analysis was performed to report the associated risk of mortality and WoC within 72-hours for patients with and without MC. This was reported with an odds ratio (OR) with 95 % confidence intervals (CI) and a *p* < 0.05 was considered statistically significant. All analyses were performed with IBM SPSS Statistics for Windows (Version 28, IBM Corp., Armonk, NY).

## Results

### Demographics of trauma patients with metastatic cancer (Table 1, Table 2)

Of 43,826 trauma patients, 62 had MC with isolated torso trauma and underwent emergency surgery. After propensity-score matching, 39 trauma patients with MC were compared to 78 trauma patients without MC who also underwent emergent surgery. There was no difference in age, sex, ISS, vitals on admission, comorbidities, mechanism of injury and need for blood transfusion. All injuries were similar between both groups except MC patients had a lower rate of colon injuries compared to non-MC patients (2.6 % vs 17.7 %, *p* = 0.020). In the MC group, the median age was 61, the most common comorbidity was hypertension (30.8 %), and the most common injury was to the lungs (43.6 %).

**Table 1:** Demographics of 1:2 propensity matched trauma patients undergoing emergent thoracic or abdominal operations with and without metastatic cancer.

	Patients without MC	Patients with MC	
Characteristic	(*n* = 78)	(*n* = 39)	*p*-value
Age, year, median (IQR)	63 (22)	61 (22)	1.000
Male, n (%)	32 (40.5 %)	(41.0 %)	0.957
Glasgow coma score, n (%)			0.911
14–15	47 (59.5 %)	24 (61.5 %)	
9–13	8 (10.1 %)	3 (7.7 %)	
3–8	24 (30.4 %)	12 (30.8 %)	
Injury severity score, n (%)			0.993
ISS ≤9 or less	19 (24.1 %)	9 (23.1 %)	
ISS 10–15	9 (11.4 %)	5 (12.8 %)	
ISS 16–24	9 (11.4 %)	4 (10.3 %)	
ISS ≥25	42 (53.2 %)	21 (53.8 %)	
Vitals on admission, n (%)			
Hypotensive (SBP <90 mmHg)	14 (17.7 %)	8 (20.5 %)	0.714
Tachycardic, HR >120 bpm	9 (11.4 %)	4 (10.3 %)	0.853
Tachypneic, RR >22	23 (29.1 %)	12 (30.8 %)	0.853
Comorbidities, n (%)			
Anticoagulant use	2 (2.5 %)	0 (0 %)	0.316
Congestive heart failure	1 (1.3 %)	0 (0 %)	0.480
COPD	1 (1.3 %)	0 (0 %)	0.480
Diabetes	12 (15.2 %)	5 (12.8 %)	0.730
Hypertension	30 (38.0 %)	12 (30.8 %)	0.422
Myocardial infarction	1 (1.3 %)	0 (0 %)	0.480
Smoking	10 (12.7 %)	5 (12.8 %)	0.980
pRBC transfused, n (%)	34 (43.0 %)	17 (43.6 %)	0.955
Penetrating mechanism, n (%)	15 (19.0 %)	8 (20.5 %)	0.844
Blunt mechanism, n (%)	65 (82.3 %)	32 (33.0 %)	0.976
Operation by Organ System, n (%)			
Cardiovascular	8 (10.1 %)	3 (7.7 %)	0.669
Hepatobiliary	10 (12.7 %)	7 (17.9 %)	0.441
Respiratory	22 (27.8 %)	15 (38.5 %)	0.242
Gastrointestinal	41 (51.9 %)	17 (43.6 %)	0.396

ISS = injury severity score; MC = metastatic cancer;SBP = systolic blood pressure; HR = heart rate; bpm = beats per minute RR = respiratory rate; pRBC = packed red blood cells.

**Table 2:** Injuries of 1:2 propensity matched trauma patients undergoing emergent thoracic or abdominal operations with and without metastatic cancer.

	Patients without MC	Patients with MC	
Injuries, n (%)	(n = 78)	(n = 39)	p-value
Rib fracture	26 (32.9 %)	14 (35.9 %)	0.747
Heart	7 (8.9 %)	3 (7.7 %)	0.830
Lung	28 (35.4 %)	17 (43.6 %)	0.391
Diaphragm	8 (10.1 %)	5 (12.8 %)	0.660
Stomach	1 (1.3 %)	1 (2.6 %)	0.607
Liver	14 (17.7 %)	6 (15.4 %)	0.750
Gallbladder	2 (2.5 %)	0 (0 %)	0.316
Pancreas	5 (6.3 %)	0 (0 %)	0.108
Spleen	19 (24.1 %)	7 (17.9 %)	0.452
Kidney	5 (6.3 %)	1 (2.6 %)	0.381
Small intestine	8 (10.1 %)	1 (2.6 %)	0.145
Colon	14 (17.7 %)	1(2.6 %)	**0.020**
Rectum	3 (3.8 %)	0 (0 %)	0.218
Pelvic fracture	9 (11.4 %)	2 (5.1 %)	0.271
Bladder	2 (2.5 %)	0 (0 %)	0.316

Bold indicate statistical significance (p<0.05).

### Clinical outcomes for trauma patients with and without metastatic cancer (Table 3)

Compared to patients without MC, patients with MC had a higher rate of mortality (38.5 % vs 15.2 %, *p* = 0.005) and WoC within 72 h (12.8 % vs 1.3 %, *p* = 0.007). Both cohorts had similar rates of all complications (all *p* > 0.05) and a similar median hospital LOS (12 days, *p* = 0.689) and ventilator days (10.2 vs 8.0 days, *p* = 0.724).

**Table 3:** Outcomes of 1:2 propensity matched trauma patients undergoing emergent thoracic or abdominal operations with and without Metastatic cancer.

	Patients without MC	Patients with MC	
Outcome	(n = 78)	(n = 39)	p-value
LOS, days, median (IQR)	12 (13)	12 (11)	0.689
Ventilator, days, mean (SD)	10.25 (1.38)	8.00 (2.60)	0.724
Complications, n (%)			
Acute kidney injury	1 (1.3 %)	2 (5.1 %)	0.210
ARDS	2 (2.5 %)	1 (2.6 %)	0.992
Cardiac arrest	3 (3.8 %)	2 (5.1 %)	0.736
Myocardial infarction	1 (1.3 %)	0 (0 %)	0.480
CLABSI	0 (0 %)	1 (2.6 %)	0.153
CAUTI	1 (1.3 %)	0 (0 %)	0.480
Ventilator associated pneumonia	6 (7.6 %)	0 (0 %)	0.077
Sepsis	0 (0 %)	1 (2.6 %)	0.153
Superficial SSI	1 (1.3 %)	0 (0 %)	0.480
Deep SSI	1 (1.3 %)	1 (2.6 %)	0.607
Deep vein thrombosis	3 (3.8 %)	1 (2.6 %)	0.728
Pulmonary embolism	2 (2.5 %)	0 (0 %)	0.316
Stroke	2 (2.5 %)	0 (0 %)	0.316
Pressure ulcer	3 (3.8 %)	0 (0 %)	0.218
Unplanned ICU admission	5 (6.3 %)	3 (7.7 %)	0.782
Unplanned intubation	1 (1.3 %)	0 (0 %)	0.480
Unplanned return to OR	2 (2.6 %)	0 (0 %)	0.326
Discharged/Transferred to SNF			
Withdrawal of care ≤72 Hours	1 (1.3 %)	5 (12.8 %)	0.007
Mortality, n (%)	12 (15.2 %)	15 (38.5 %)	0.005

LOS = length of stay; IQR = interquartile range; UTI = urinary tract infection; CLABSI = central line associated bloodstream infection; SSI = surgical site infection; ARDS = acute respiratory distress syndrome; OR = operating room; ICU = intensive care unit; SNF = skilled nursing facility.

### Analysis for risk of mortality and withdrawal of care within 72 h (Table 4)

Patients with MC had a higher associated risk of death during index hospitalization compared to patients without MC (OR 3.49, CI 1.43–8.51, *p* = 0.006), as well as a higher associated risk of WoC within 72-hours (OR 11.47, CI 1.29–101.93, *p* *=* 0.029). When performing a sensitivity analysis by adding WoC in a multivariable logistic regression model, the associated risk of death continued be strong for trauma patients with MC (OR 2.17, CI 1.28–3.64, *p* = 0.001).

**Table 4:** Logistic regression analysis for 1:2 propensity matched metastatic vs. non-metastatic-cancer trauma patients undergoing emergent thoracic or abdominal operations.

Risk factor	OR	CI	p-value
Mortality	3.49	1.43–8.51	0.006
Withdrawal of care within 72-hours	11.47	1.29–101.93	0.029

## Discussion

In the past decade, the incidence of MC has increased, thus more trauma patients may present with this diagnosis. This national analysis evaluated the clinical outcomes of trauma patients with and without MC undergoing emergent thoracic or abdominal surgery and found that patients with MC had a higher rate and associated risk of death and WoC within 72 h. To our knowledge, this is the first investigation directly comparing the clinical outcomes of a nationwide cohort of patients with MC to patients without MC undergoing emergent surgery.

Emergency general surgery (EGS) patients with MC undergoing emergent surgery have a mortality rate of nearly 20 % and a complication rate >40 % [20]. This analysis spanning three years of data for trauma patients with MC found they had an even higher mortality rate (nearly 40 %) than EGS patients. Trauma patients without MC requiring emergent surgery had a mortality rate of less than half of those with MC and a rate more consistent with EGS patients (20 % in the literature) [21]. MC may be especially lethal for trauma patients for several reasons. The immune system in MC patients is more susceptible to infections. This may be due to ongoing systemic chemotherapy or an immunosuppressive state that is caused by widespread cancer itself [22]. In fact, the risk of developing pneumonia is four-fold higher for MC patients undergoing surgery compared to patients without cancer [12,23]. Infectious complications have been demonstrated to increase the risk of death in patients with cancer by nearly three-fold [24]. MC patients also have a 12-fold increased risk of developing thrombotic and embolic events given their hypercoagulable state [13,25]. Interestingly, our findings show no significant difference in complications between trauma patients with and without MC. This may be due to survivor bias as nearly half the patients in the MC group died or underwent WoC within 72-hours which may have not permitted enough time for the development or identification of a complication.

Cancer patients in the elective setting have benefited from multidisciplinary approaches to care including development of specific conferences (e.g., tumor board) to align care providers and discuss optimal treatment paradigms [26]. Patients undergoing emergency surgery may also benefit from a multidisciplinary approach to their care, although this may be more difficult given time constraints. In further hindrance, only a small minority of these patients have had prior discussions regarding goals of care documented in the electronic medical record and/or physician orders for life sustaining treatment (POLST). The latter may prove especially helpful as it is a legally binding order that must be utilized [27]. In addition, there has been growing interest in developing a multidisciplinary team to help tackle emergent conditions including emergency surgery in cancer patients [28]. This has led to the inception of “acute oncology” and has been used to help inform, manage, and provide care for MC patients presenting with bowel perforation or obstruction [29]. Since its inception, treatment of metastatic disease has been progressively improving, such that life-saving interventions should not be withheld unless patients have clearly defined documentation to withholding surgical intervention [30]. Although some trauma patients presenting in extremis may not have enough time or be lucid enough to participate in detailed informed consent, this is not universally true for all trauma patients [17,18]. For example, a MC trauma patient with a Glasgow coma scale score of 15 and concern for hollow viscous injury requiring an emergent operation would be able to participate and benefit from these discussions.

Physicians have traditionally associated quality of care for MC with underuse of treatment, including emergent surgery, instead of considering the strong possibility that treatment overuse may lead to worse quality of life and care for MC patients [31]. Furthermore, there is large variability in perspective and lack of knowledge regarding proper postoperative management and resources for cancer patients, ultimately contributing to the higher mortality rates in MC patients [32]. An important consideration is the potential provider bias towards MC patients following trauma. Providers may be more inclined to discuss goals of care and WoC with MC patients due to their pre-existing life-limiting disease. This bias could influence decision-making processes, possibly leading to a higher rate of WoC discussions and decisions within the first 72 h. Recognizing this bias is important for ensuring that MC patients receive equitable and patient-centered care. Similar to non-trauma patients with MC, our study found that trauma patients with MC not only have a higher mortality rate after emergency surgery, but also have a >10-fold higher risk of WoC shortly after emergency surgery which may suggest there is a role for non-operative palliative options in select patients [17]. Despite having a higher risk for poor outcomes and more comorbidities compared to other patient populations, a palliative care consult is rare for trauma patients [33,34]. In both trauma and non-trauma patients, involvement of palliative care can reduce symptom burden as well as improve quality of life and resource utilization [[35], [36], [37], [38], [39], [40]]. Hence, a palliative care consult should be sought for select trauma patients with MC to help aid in discussions and identify goals of care, upon arrival to the trauma center.

As a large retrospective database study, there are inherent limitations to this study including selection and reporting bias, and coding errors. Given the acuity of an emergent operation and trauma activation, it may be challenging to make an accurate assessment of the extent of metastatic disease by the trauma surgeon. This is reflected in a lack of granular information in the database regarding the specific type of MC, location(s) of spread (i.e., liver, brain, bone etc.). Additionally, the TQIP database lacks baseline Eastern Cooperative Oncology Group (ECOG) performance status amongst other pertinent missing variables such as pertinent radiographic findings and TNM staging. There is also a lack of information regarding the extent of palliative care involvement and the presence of an advance directive documented in the database. Finally, we are unable to assess causality due to the retrospective nature of this study. However, the results of our studies emphasize the need for further exploration on the relationship between metastatic location and outcomes, and the role of palliative care in the assistance of these patients undergoing emergent surgeries.

## Conclusions

Trauma patients with MC suffer an increased risk of mortality and are more likely to undergo withdrawal of care compared to similarly matched patients without MC. Efforts to establish goals of care as an outpatient (e.g., POLST) and/or early palliative care services following traumatic injury should be considered for patients with MC. In light of the limitations associated with registry databases, future research should focus on a prospective cohort study to gather more granular data on MC trauma patients including detailed information on the types and locations of metastases, baseline performance status, and the extent of palliative care involvement. Additionally, integrated qualitative interviews with providers to better understand decision-making processes regarding goals of care and withdrawal of care would be helpful.

## Funding

This research did not receive any specific grant from funding agencies in the public, commercial, or not-for-profit sectors.

## Ethics approval

This study was deemed exempt by our Institutional Review Board and a waiver of informed consent granted as it utilizes a national deidentified database.

## CRediT authorship contribution statement

**Matthew Nguyen:** Conceptualization, Data curation, Formal analysis, Methodology, Writing – original draft, Writing – review & editing. **Jeffry Nahmias:** Conceptualization, Methodology, Writing – original draft, Writing – review & editing. **Oliver S. Eng:** Conceptualization, Data curation, Methodology, Writing – original draft, Writing – review & editing. **Maheswari Senthil:** Formal analysis, Methodology, Writing – original draft, Writing – review & editing. **Cristobal Barrios:** Formal analysis, Methodology, Writing – original draft, Writing – review & editing. **Matthew Dolich:** Formal analysis, Methodology, Writing – original draft, Writing – review & editing. **Michael Lekawa:** Conceptualization, Methodology, Writing – original draft, Writing – review & editing. **Areg Grigorian:** Conceptualization, Data curation, Formal analysis, Methodology, Supervision, Writing – original draft, Writing – review & editing.

## Declaration of competing interest

The authors report no conflicts of interest.
